# Finding a Compatible Partner: Self-Incompatibility in European Pear (*Pyrus communis*); Molecular Control, Genetic Determination, and Impact on Fertilization and Fruit Set

**DOI:** 10.3389/fpls.2019.00407

**Published:** 2019-04-16

**Authors:** Hanne Claessen, Wannes Keulemans, Bram Van de Poel, Nico De Storme

**Affiliations:** ^1^ Laboratory for Plant Genetics and Crop Improvement, Division of Crop Biotechnics, Department of Biosystems, KU Leuven, Leuven, Belgium; ^2^ Laboratory for Molecular Plant Hormone Physiology, Division of Crop Biotechnics, Department of Biosystems, KU Leuven, Leuven, Belgium

**Keywords:** *Pyrus communis*, gametophytic self-incompatibility, fertilization, fruit set, S-RNase, SFBB

## Abstract

*Pyrus* species display a gametophytic self-incompatibility (GSI) system that actively prevents fertilization by self-pollen. The GSI mechanism in *Pyrus* is genetically controlled by a single locus, i.e., the S-locus, which includes at least two polymorphic and strongly linked S-determinant genes: a pistil-expressed *S-RNase* gene and a number of pollen-expressed *SFBB* genes (S-locus F-Box Brothers). Both the molecular basis of the SI mechanism and its functional expression have been widely studied in many Rosaceae fruit tree species with a particular focus on the characterization of the elusive *SFBB* genes and S-RNase alleles of economically important cultivars. Here, we discuss recent advances in the understanding of GSI in *Pyrus* and provide new insights into the mechanisms of GSI breakdown leading to self-fertilization and fruit set. Molecular analysis of S-genes in several self-compatible *Pyrus* cultivars has revealed mutations in both pistil- or pollen-specific parts that cause breakdown of self-incompatibility. This has significantly contributed to our understanding of the molecular and genetic mechanisms that underpin self-incompatibility. Moreover, the existence and development of self-compatible mutants open new perspectives for pear production and breeding. In this framework, possible consequences of self-fertilization on fruit set, development, and quality in pear are also reviewed.

## Introduction

**Figure 1:**
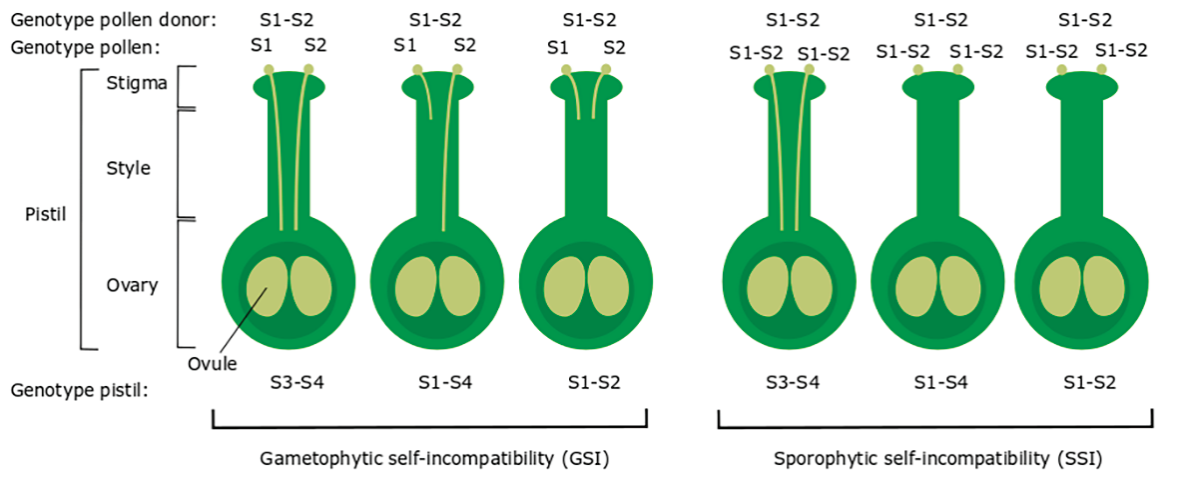
Illustration of the genetic basis of gametophytic SI (GSI) and sporophytic SI (SSI). In GSI, the pollen carries one of two S-haplotypes of the pollen parent (pollen donor), in this case either S1 or S2. If the S-haplotype of the pollen matches one of the two S-haplotypes of the pistil, the pollen is rejected after growing through approximately one-third of the style. In SSI, the pollen S-haplotype is determined by both S-haplotypes of the pollen parent. If the S-haplotype of the pollen donor matches one or both S-haplotypes of the pistil, the pollen is rejected and will not germinate. This figure represents SSI in case of co-dominance between S-alleles. In SSI of certain species, the presence of dominant/recessive alleles can result in more complex patterns of compatibility/incompatibility.

Self-incompatibility (SI) refers to all genetic mechanisms in flowering plants that prevent self-fertilization through the recognition and rejection of self-pollen by the style of a flower (DeNettancourt, 1977). SI is generally classified into two types: heteromorphic and homomorphic SI. The heteromorphic SI system includes distyly and tristyly and inhibits self-fertilization through the production of more than one morphological flower type. In contrast, homomorphic SI inhibits self-fertilization through genetic or biochemical mechanisms that operate regardless of flower morphology (Charlesworth, 2010; Orević et al., 2014). There are two main types of homomorphic SI: gametophytic self-incompatibility (GSI) and sporophytic self-incompatibility (SSI) (Kao and Huang, 1994) (Figure 1). In GSI, the genotype of the haploid pollen itself (gametophyte) determines its incompatibility type, while in SSI, the genotype of the diploid parental plant (sporophyte) that acts as the pollen donor determines the incompatibility type (Hiscock and Tabah, 2003). GSI is considered the most prevalent SI system in the plant kingdom and occurs in Solanaceae, Rosaceae, and Plantaginaceae (Franklin-Tong and Franklin, 2003a).

European pear (*Pyrus communis*) exhibits an RNase-based gametophytic self-incompatibility system (Sassa et al., 1992). This system is genetically controlled by a single locus, named the S-locus, which includes at least two polymorphic genes that are tightly linked: a pistil-expressed gene and one or several pollen-expressed genes (Entani et al., 2003; Kao and Tsukamoto, 2004). The pistil S-gene encodes an S-RNase, which is highly expressed in the style and catalyzes degradation of RNA (Sassa et al., 1992, 1996; Zhou et al., 2016). In *Pyrus* species, the pollen S-determinant is proposed to consist of multiple *F-box* genes, called *SFBBs* (S-locus F-Box Brothers) (Sassa et al., 2007). Across all *Pyrus communis* genotypes, there exists great variability in the S-locus haplotype, i.e., as reflected by the allelic variability in both the pollen- and pistil-expressed genes. For fertilization to take place, the S-haplotype of the pollen grain must differ from the two S-haplotypes of the diploid pistil, otherwise the growth of the pollen tube is arrested after it has migrated through about one-third of the length of the style (DeNettancourt, 1977; Franklin-Tong and Franklin, 2003b). The GSI mechanism therefore inhibits specific hybridizations between *Pyrus communis* genotypes that carry the same S-haplotypes.

Self-incompatibility and other reproductive strategies that promote outcrossing, such as dioecy, dichogamy, and male sterility, are considered to have played an important role in the success of angiosperms. By stimulating outbreeding, SI promotes gene flow and associated genetic diversity on which selection can act (DeNettancourt, 1977). However, in crop cultivation, SI often forms a major obstacle. In monocultures, for example, where compatible mates and, therefore, cross-pollination events are also limited, SI leads to a reduced production of seeds and/or fruits (Miller and Gross, 2011). In addition to this, cross-pollination is often strongly hampered by adverse weather conditions and a low attractiveness of flowers to insect pollinators (Quinet et al., 2016), causing great year-to-year variation in pollination efficiency. For pear and other fruit trees, this can lead to unpredictable fruit set and financial insecurity for the grower, even when compatible pollinizers have been planted in the orchard. In plant breeding, SI strongly limits the range of possible mating combinations and hybridization events. In outcrossing species, it is very difficult to combine desirable traits of two incompatible parents through simple cross-pollination. Because of this, introduction of self-compatibility has become a major objective in many fruit tree species. On the one hand, the use of self-compatible lines will broaden the available options for increasing genetic variability in crop improvement. On the other hand, at the level of crop production, it will avoid the need for specific pollinizer cultivars, and result in a more uniform fruit set which is less influenced by environmental fluctuations (Tehrani and Brown, 2010; Cachi and Wünsch, 2014).

Spontaneous induction of self-compatibility in SI species has been found to occur in nature, as, for example, shown by the broad variety of self-compatible genotypes in *Prunus* (Sawamura et al., 2013; Cachi and Wünsch, 2014). In *Pyrus*, however, only a small number of spontaneous self-compatible mutants have been identified (Sawamura et al., 2013; Wu et al., 2013), most of which belong to the species *Pyrus pyrifolia* (Japanese pear – syn: *Pyrus serotina*). These self-compatible *Pyrus* genotypes are most often the result of a pistil-part mutation, that either leads to a non-functional S-RNase or that reduces its expression in the style (Li et al., 2009; Sawamura et al., 2013; Wu et al., 2013).

In this review, we present the latest insights into the mechanism of SI in *Pyrus* and specifically in *Pyrus communis*. We present recent advances on the genetic determination and molecular control of SI, and additionally discuss breakdown of self-incompatibility and its impact on fruit development and final fruit set in pear.

## The Genetic Control of GSI in *Pyrus*

### The S-locus

**Figure 2:**
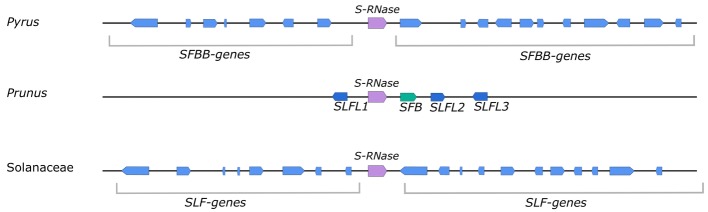
Putative S-locus structure of *Pyrus*, *Prunus*, and Solanaceae species. In all cases, the S-locus contains an *S-RNase* gene (purple arrow), which acts as the pistil S-determinant. For *Pyrus* and Solanaceae species, this *S-RNase* gene is surrounded by a large number of *SFBB/SLF* genes (blue arrows) which are proposed to make up the pollen S-determinant. For *Pyrus*, the expected number of *F-box* genes (*SFBB* genes) is approximately 18–20, which is comparable to the observed number of F-box genes (*SLF* genes) in *Petunia* (Solanaceae). It is expected that the size, orientation, and position of these *F-box* genes relative to the *S-RNase* gene are variable between S-haplotypes. In *Prunus*, the pollen S-determinant is the *SFB* gene (green arrow) that is located closest to the *S-RNase*. The three surrounding *SLFL* genes (dark blue arrows) are relatively closely related to the *SFBB* and *SLF* genes of *Pyrus* and Solanaceae, respectively. It is suggested that they function as the general inhibitor in the *Prunus* SI system. Figure based on DeFranceschi et al. (2012).

Genetic control of GSI in *Pyrus* is mainly situated at and regulated by the S-locus. In addition, however, some “modifier” genes that are not linked to the S-locus are also known to play a role in the functioning of GSI. These “modifier” genes, like the SKK1 protein, often interact with the proteins coded by the S-locus (Wu et al., 2013; Xu et al., 2013). The *Pyrus* S-locus consists of a single *S-RNase* gene surrounded by multiple *F-box* genes, which are referred to as *SFBB* genes (Sassa et al., 2007). A schematic overview of the S-locus in *Pyrus*, *Prunus*, and Solanaceae is presented in Figure 2. In *Pyrus*, the S-locus is positioned at the subtelomeric region of chromosome 17 (Maliepaard et al., 1998; Yamamoto et al., 2002). A first prediction of the genomic structure of the S-locus of pear was made in Japanese pear (*Pyrus pyrifolia)* using BAC cloning and sequencing (Sassa et al., 2007). This work revealed for the first time the presence of multiple *SFBB* genes that surround the *S-RNase*. Sequence comparison of the genomic regions surrounding the *S2*- and *S4-RNases* revealed that the S-haplotypes in pear can show significant variation in the position and orientation of the *SFBB* genes relative to the *S-RNase* gene (Figure 2) (Okada et al., 2011). As yet, it is still unclear how many SFBB genes are involved in the GSI system. However, based on findings in apple (*Malus domestica*), the currently estimated number of SFBB genes is 17–19 (Pratas et al., 2018). In addition, the S-locus sequence in pear was also found to contain numerous transposon-like sequences which are proposed to generate polymorphisms among S-haplotypes, and which likely contribute to the suppression of meiotic recombination between the *S-RNase* and *SFBBs* (Okada et al., 2011). In contrast, the S-locus of *Prunus* species consists of a single *S-RNase* gene and a single *SFB* (S-haplotype specific F-box) gene, which determines pollen specificity. The specific S-genes are surrounded by three *SLFL* (S-locus F-box-like) genes, whose function is still not clarified (Figure 2) (Entani et al., 2003; Romero et al., 2004; Matsumoto et al., 2008).

Recombination suppression in the S-locus region is essential because the pistil S- and pollen S-genes must inherit as one single unit in order to maintain the functionality of the SI system (Roalson and McCubbin, 2003). This recombination-suppressed region is predicted to be much larger in *Malus* and *Pyrus* compared to that of *Prunus* species (Matsumoto and Tao, 2016a). The size prediction of the S-locus of *Prunus* was based on the observation that the region around the *S-RNase* gene exhibits extreme sequence diversity and contains transposable elements, which is in contrast to the high colinearity and presence of conserved genes outside this region (Entani et al., 2003). The size of this region was estimated to be at least 1 Mb in *Malus* and at least 649 kb in *Pyrus* species, compared to merely 70 kb in *Prunus* species (Entani et al., 2003; Okada et al., 2011; Matsumoto and Tao, 2016a).

### The *S-RNase* Gene

The pistil determinant of GSI in Rosaceae was first identified in Japanese pear (*Pyrus pyrifolia*) as a stylar RNase which shows high similarity with the previously identified S-RNase in Solanaceae (Sassa et al., 1992). In Solanaceae, a stylar glycoprotein with ribonuclease activity acts as the female S-determinant of GSI, because it is abundantly expressed in the style of self-incompatible *Nicotiana alata* and co-segregates with the observed S-phenotypes (Bredemeijer and Blaas, 1981; Anderson et al., 1986; McClure et al., 1989). This RNase belongs to the RNase T2 family and is therefore named S-RNase (McClure et al., 1989). Members of the RNase T2 family have a wide range of cytotoxic functions: from rRNA degradation to direct induction of cell death (Luhtala and Parker, 2010). In *Pyrus,* functional analysis of self-compatible genotypes carrying spontaneous pistil-part mutations confirmed that the S-RNase is indeed the female S-determinant (Huang et al., 1994; Murfett et al., 1994; Sassa et al., 1997; Sanzol, 2009a). Importantly, studies in Solanaceae have shown that the RNase activity of the S-RNase is essential for pistil S-function (Huang et al., 1994; Kowyama et al., 1994; Matsumoto and Tao, 2016a). Based on this, the mechanism of GSI in *Pyrus* was suggested to act *via* RNase-based degradation of the cellular RNA in germinating pollen tubes, thereby causing inhibition of pollen tube growth (Huang et al., 1994; Murfett et al., 1994). However, more recent evidence in *Pyrus* revealed that incompatible pollen tubes exhibit several typical characteristics of programmed cell death (PCD) during SI reaction (Liu et al., 2007; Wang et al., 2009, 2010; DeFranceschi et al., 2012). Also, a recent study demonstrated that the S-RNase of *Pyrus bretschneideri* (PbrS-RNase) directly interacts with PbrActin1 and induces cross-linkage between actin filaments of incompatible pollen tubes (Chen et al., 2018b). Similar actin-binding properties are observed for other members of the T2-RNase family, for example for ACTIBIND, a T2-RNase produced by *Aspergillus niger* B1 (CMI CC 324626) (Roiz et al., 2000). This interaction, however, was shown to be non-S-allele specific and independent of RNase activity. Overall, these observations demonstrate that RNA degradation might not be the only process involved in the inhibition of pollen tube growth and indicate that the S-RNase may have other targets than cellular RNA in the pollen tube (Chen et al., 2018b).

**Figure 3:**
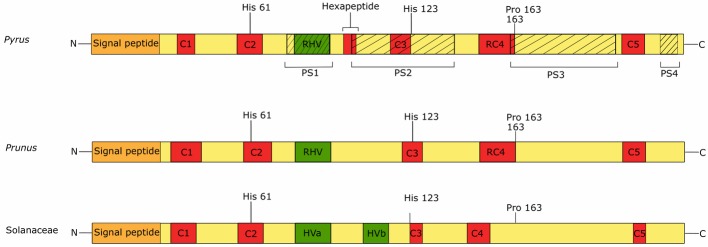
Predicted *S-RNase* protein sequences of *Pyrus*, *Prunus*, and Solanaceae species. All sequences contain a signal peptide (orange box) and five conserved regions (C1–C5, red boxed). Conserved regions C2 and C3 each contain a histidine residue which is essential for the ribonuclease activity of the protein. Conserved region RC4 is specific for Rosaceae and is present in *Pyrus* and *Prunus*, while C4 is specific for Solanaceae. This region contains a proline residue which is involved in the interaction with actin. Rosaceae genera *Pyrus* and *Prunus* have a single hypervariable region (green boxes), namely the RHV (Rosaceae hypervariable region). Solanaceae species have two hypervariable regions, of which HVa corresponds to the RHV region. Four positively selected regions (PS1–4, hatched boxes) are identified in the S-RNase protein sequence of *Pyrus*.

The structure of the *S-RNase* gene of *Pyrus* consists of five small, conserved regions (C1 to C5) and one hypervariable region (the Rosaceae hypervariable region, RHV), which contains the single intron that is highly polymorphic in length. The hypervariable region of the Rosaceae *S-RNase* gene corresponds to one of the two hypervariable regions of the Solanaceaous *S-RNase*, namely HVa (Matsuura et al., 2001). Specifically in the Maloidae, a highly conserved hexapeptide region (IIWPNV) is located immediately downstream of the RHV region. The DNA sequence encoding this hexapeptide has frequently been used for the development of consensus primers for PCR-based S-genotyping (DeFranceschi et al., 2012). S-RNase genotyping by PCR is commonly used in Rosaceae species to determine incompatibility relations between cultivars, often in combination with field-controlled pollination assays (Zuccherelli et al., 2002; Larsen et al., 2016; Herrera et al., 2018). Figure 3 displays a schematic representation of the protein sequence of the S-RNase of *Pyrus, Prunus*, and Solanaceae. All conserved regions in the Rosaceae S-RNases show high sequence similarity with the conserved regions of the S-RNase in Solanaceae, except for C4, which was therefore renamed “Rosaceae Conserved Region 4” (RC4) (Zisovich et al., 2004). Conserved regions C1, RC4, and C5 are thought to be involved in the stabilization of the enzyme structure due to the high number of hydrophobic amino acids (Ida et al., 2001; Matsuura et al., 2001; Zisovich et al., 2004). RC4 and more specifically the proline at position 156 have been suggested to be responsible for the interaction with actin in *Pyrus bretschneideri* (Chen et al., 2018b). Analogous protein domain structures were also found in ACTIBIND and RNASET2, members of the RNase T2 family in fungi and humans that also bind actin to induce cross-links between actin filaments (Roiz et al., 2000; Chen et al., 2018b). The C2 and C3 S-RNase regions contain conserved catalytic histidine residues that play an important role in RNase activity (Horiuchi et al., 1988; Kao and Huang 1994). The hypervariable RHV region between C2 and C3 is located at the protein surface and was therefore long thought to underpin selective interaction between the S-RNase and the pollen S-determinant (Matton et al., 1997). However, studies in European pear (*Pyrus communis*) revealed that PcS106-pollen tube growth is not inhibited by the PcS116-RNase, although PcS106 and PcS116 S-RNase have identical deduced amino acid sequences in their RHV region. This suggests that the RHV region is not sufficient for selective interaction with the pollen S-protein (Zisovich et al., 2004). Four other protein regions (PS1–PS4) might be more important for the selective interaction with the pollen S-determinants. These regions show an excess amount of non-synonymous amino acid substitutions over synonymous substitutions (K_a_/K_s_), suggesting high sensitivity to positive selection (Ishimizu et al., 1998; Zisovich et al., 2004). Since a single protein is unlikely to interact with all four PS regions, the common hypothesis is that multiple proteins simultaneously interact with the S-RNase to determine the SI response in pear (Matsuura et al., 2001; Vieira et al., 2010).

### The Pollen-S Gene(s)

The pollen S-determinant of GSI was identified almost 15 years after the discovery of the pistil S-determinant. The first promising candidate was identified in *Antirrhinum hispanicum*, a member of the Plantaginaceae. Sequencing of a 64-kb region around the *S2-RNase* gene revealed the presence of an F-box gene, named *AhSLF-S2* (*A. hispanicum* S-locus F-box of S2-haplotype) (Lai et al., 2002; Zhou et al., 2003). F-box proteins contain at least one F-box domain and are one of three components of the SCF complex (SkpI, Cullin and F-box protein complex), which mediates targeted protein degradation *via* the ubiquitin-26S proteasome. In this complex, the F-box protein specifically recognizes the target protein and thereby contributes to the specificity of SCF (Kipreos and Pagano, 2000; Smalle and Vierstra, 2004). As predicted for the pollen S-determinant, *AhSLF-S2* is polymorphic, linked to the S-locus, and specifically expressed in pollen (Qiao et al., 2004). Moreover, the *Ah*SLF-S2 F-box protein was found to physically interact with S-RNases in a non-allele-specific way, confirming its role as pollen S-determinant (Qiao et al., 2004). In Rosaceae, S-linked F-box genes were first reported as candidate for the pollen S-determinant in *Prunus* (Entani et al., 2003; Ushijima et al., 2003; Sonneveld et al., 2005). In *Prunus mume* (Japanese apricot), the genomic region surrounding the *S-RNase* gene contains at least four F-box genes, but only the F-box gene closest to the *S-RNase* gene was found to encode the pollen S-determinant (Entani et al., 2003). This specific F-box gene was termed the *SFB* (S-haplotype-specific F-box), whereas the other three were named *SLFLs* (S-locus F-box like) (Figure 2) (Entani et al., 2003).

In *Pyrus* and *Malus,* emerging evidence suggests the presence of multiple related F-box genes within the S-locus, which are referred to as *SFBB*s (S-locus F-box brothers) (Zhou et al., 2003). Using a BAC library from the apple cultivar Florina, two *SFBB* genes were identified in the 317-kb sequence around the S9-RNase: *MdSFBB^9-a^* and *MdSFBB^9-b^* (Sassa et al., 2007). Using primers derived from these *MdSFBB* sequences, the same study isolated six cDNA sequences from pollen of the Japanese pear (*Pyrus pyrifolia*) cultivar Kosui (*Pp*S4-*Pp*S5): *PpSFBB^4-α^, PpSFBB^4-β^*, *PpSFBB^4-γ^, PpSFBB5^5-α^, PpSFBB^5-β^*, *PpSFBB^5-γ^*. Cleaved amplified polymorphic sequence (CAPS) analysis of these *PpSFBB* genes confirmed their linkage with the *S-RNase* gene and also pollen-specific expression (Sassa et al., 2007). This study therefore showed that the S-haplotypes of apple (M. domestica) and Japanese pear (*Pyrus pyrifolia*) contain multiple copies of the *SFBB* gene and postulated that these *SFBB* genes are convincing candidates to act as the pollen S-determinant in *Malus* and *Pyrus* (Sassa et al., 2007). The *PpSFBB^γ^* gene was further characterized in other S-haplotypes of Japanese pear and, based on its inherent variability, used for the development of a molecular S-genotyping assay (Kakui et al., 2007). A later study in Japanese pear in which a 240-kb region surrounding the *S4-RNase* was sequenced resulted in the identification of a new pollen-specific F-box gene, named *S^4^F-box0* (F4-haplotype F-box protein gene), that differs from the previously identified *PpSFBB^4α-γ^* (Okada et al., 2008). The self-compatible (SC) “Osa Nijisseiki” (*Pp*S2/*Pp*S4^sm^) is a natural stylar-part mutant (sm) derived from “Nijisseiki” (*Pp*S2/*Pp*S_4_) that lacks pistil S function but retains pollen S function (Okada et al., 2011). The S4^sm^-haplotype of this mutant has a 236-kb deletion in the S-locus, causing loss-of-function of both the *S4-RNase* and *S4F-box0* genes. These findings suggest that the pollen S-determinant for the S4-haplotype is not conferred by the *S4F-box0,* and should be located outside the region spanning 48 kb upstream to 188 kb downstream of *S4-RNase* (Okada et al., 2008). However, an earlier study showed that S4^sm^ pollen are not only rejected by styles carrying the S4-haplotype, but also by S1-haplotye styles, while still being compatible with styles of other non-self S-haplotypes. This suggests that S4F-box0 specifically recognizes S1-RNase (Okada et al., 2011). This observation is consistent with the non-self-recognition model proposed for the GSI system in *Pyrus* (Kubo et al., 2010; DeFranceschi et al., 2012). In this model, each SFBB F-box protein specifically recognizes only one or a few S-RNases, and multiple SFBB work together to recognize non-self S-RNases and mark them for degradation. Okada et al. (2011) sequenced an additional 6 *SFBB* genes in a 378-kb region around *S2-RNase* (*PpSFBB^4 u1-u4, 4 d1-d2^*) and 10 *SFBB* genes in a 649-kb region around the *S4-RNase* (*PpSFBB^2-u1-u5,2-d1-d5^*) of Japanese pear. Among these, *PpSFBB^4-d1^* was found to correspond to the previously identified *S4F-box0*. Similarly, in European pear (*Pyrus communis*), multiple S-locus F-box genes have been identified (Sassa et al., 2007). Six polymorphic sequences were obtained from “Abbé Fetel” (*Pc*S104–2/*Pc*S105) and ten from “Max Red Bartlett” (*Pc*S101/*Pc*S102). Hereby, *SFBB^α^*, *SFBB^β^*, and *SFBB^γ^* appeared highly homologous to *PpSFBB^α^*, *PpSFBB^β^*, and *PpSFBB^γ^*, respectively. Also, two additional *SFBB* groups were defined, *SFBB^δ^* and *SFBB^ε^*, which showed strong homology with the *MdSFBB^3-β^* and *MdSFBB^9-β^* genes of apple, respectively (DeFranceschi et al., 2011). Similarly to *Pyrus*, a multitude of *SFBB* sequences has been identified in the genomic region surrounding the *S-RNase* of other Rosaceae species. In *Prunus*, multiple *SLFL* genes have been identified at the S-locus, of which three show specific expression in pollen: *SLFL1*, *SLFL2*, and *SLFL3* (Matsumoto et al., 2008). In *M. domestica*, 10 S-haplotypes were screened for the presence of *SFBB* genes using transcriptome sequencing of anthers. For a given S-haplotype, this resulted in the identification of 17–19 *SFBB* genes (Pratas et al., 2018). A similar number of *SLF* genes (namely 16–20 *SLF* genes, classified into 18 types) was observed in *Petunia*, which also exhibits a non-self-recognition type GSI system (Matsumoto and Tao, 2016a; Pratas et al., 2018). Several SLFs thereby even appeared to target the same S-RNase (Sun and Kao, 2013; Matsumoto and Tao, 2016a). These findings suggest that the actual number of *SFBB* genes in *Pyrus* might also be around 17–20, although the exact number is still unknown. The number of pollen determinant genes currently identified in both *Petunia* and *Pyrus* are less than the number of known *S-RNase* alleles (40 or more in *Petunia*, approximately 30 in *Pyrus communis*) (Goldway et al., 2009; Sanzol, 2009a,b; Kubo et al., 2015; Larsen et al., 2016). It is therefore suggested that some SLF types in *Petunia* should interact with multiple S-RNase allelic variants, while some S-RNases may be recognized by multiple SLF types. Based on empirical data of SLF and S-RNase interaction in *Petunia* and using Monte Carlo simulation, it was estimated that 16–20 SLFs in each haplotype are adequate to recognize the vast majority of S-RNase targets (Kubo et al., 2015).

Altogether, these findings show that the S-locus of *Pyrus* contains multiple *SFBB* genes and thus shows strong similarity with the *Solanaceae* S-locus, which contains multiple *SLF* genes (Figure 2). This indicates that the SI mechanism of *Pyrus* is more similar to the SI mechanism of Solanaceae than to that of *Prunus*. However, it is currently still unknown how many *SFBB* genes in *Pyrus* are linked to the S-locus and which of these actually function as pollen S-determinant.

## The Molecular Mechanisms of Self-Recognition and Rejection in GSI of *Pyrus*

### The Mechanism of Incompatible Pollen Tube Rejection in *Pyrus*: Cellular and Biochemical Aspects

**Figure 4:**
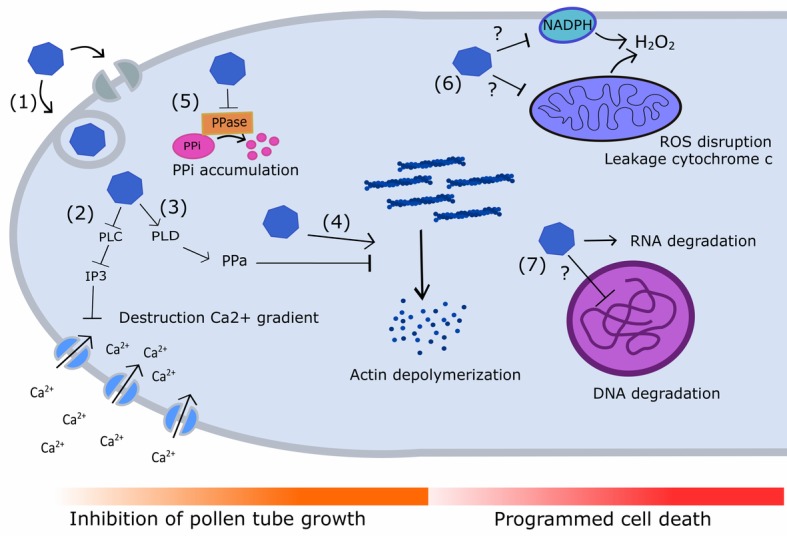
Diagram illustrating the different signaling cascades and targets underpinning S-RNase-mediated pollen tube inhibition and PCD in *Pyrus*. (1) S-RNases (blue polygon) enter the growing pollen tube by ABC transporters or *via* vesicle trafficking. Self S-RNases are not recognized by the SLF^SCF^ complex and are allowed to interact with multiple targets inside the pollen tube. (2) Self S-RNase interacts with and inhibits phospholipase C (PLC), leading to a decreased production of IP3 which in its turn reduces Ca^*2*+^ import through Ca^*2*+^ channels. This reduced Ca^*2*+^ uptake leads to the mitigation of the Ca^*2*+^ gradient in the pollen tube tip, inhibiting pollen tube growth. (3) Self S-RNases stimulate the expression of phospholipase D (PLD), which stimulates production of phosphatidic acid (PPa). PPa can temporarily delay actin depolymerization in pollen tubes, providing a first defensive mechanism against pollen tube growth inhibition. (4) However, self S-RNases can also interact directly with F-actin, causing actin depolymerization and leading to pollen tube growth inhibition. (5) Self S-RNases can physically interact with pyrophosphatases (PPases), and thereby inhibit their activity. This leads to the accumulation of inorganic pyrophosphate (PPi) which also causes reduced pollen tube growth. (6) Upon challenge with self S-RNases, the mitochondrial membrane collapses, causes leakage of cytochrome c into the cytosol and a cessation of H_2_O_2_ production. The presence of self S-RNases in the pollen tube also reduces NADPH levels, causing a decrease in plasma membrane ROS formation. As a result, tip-localized ROS accumulation is disrupted, providing another trigger for induction of PCD. (7) Challenge with self S-RNases has also been shown to cause RNA degradation and nuclear DNA degradation, two processes that are also linked to programmed cell death (PCD). Question marks denote processes in which the exact role of the S-RNase still needs to be elucidated.

Selfing or self-pollination in pears triggers a self-incompatibility (SI) reaction in the incompatible pollen tubes. This encompasses multiple cellular and biochemical changes including alterations in the actin cytoskeleton (Liu et al., 2007), swelling of mitochondria, collapse of the mitochondrial membrane potential, leakage of cytochrome c into the cytosol (Wang et al., 2009), tip-localized reactive oxygen species (ROS) and Ca^2+^ disruption (Wang et al., 2010), and degradation of nuclear DNA (Wang et al., 2009, 2010). Many of these structural and biochemical changes are characteristic of programmed cell death (PCD), which also occurs during self-pollen rejection in Papaveraceae (Bosch and Franklin-Tong, 2008; DeFranceschi et al., 2012). However, so far there is no evidence of a direct link between these processes and basic S-RNase function. Therefore, it is generally accepted that the S-RNase causes pollen tube lethality in *Pyrus* solely by degrading pollen tube RNA (McClure et al., 1990; Huang et al., 1994; DeFranceschi et al., 2012). However, increasing evidence indicates that the S-RNase may act as a trigger for biochemical processes that eventually lead up to pollen tube rejection instead of directly causing it *via* RNA degradation (Wang et al., 2010; Wang and Zhang, 2011; Qu et al., 2017). Several studies in *Pyrus* and related species showed that the S-RNase interacts with (1) F-actin (Liu et al., 2007), (2) phospholipase C (PLC) (Qu et al., 2017), and (3) pyrophosphatase (PPa) (Li et al., 2018). These interactions trigger the question whether and how overall S-RNase-based RNA degradation in selfed pollen tubes causes growth arrest. Figure 4 provides an overview of the biochemical processes linked to pollen rejection that are described below.

A first essential process in the inhibition of pollen tube growth in *Pyrus* seems to be the destruction of the Ca^2+^ gradient inside the pollen tube (Figure 4) (Qu et al., 2017). The maintenance of a tip-focused cytoplasmic Ca^2+^ gradient inside the pollen tube is essential for pollen tube growth (Holdaway-Clarke and Hepler, 2003; Qu et al., 2017). This gradient is maintained by the influx of Ca^2+^ from the style apoplast *via* membrane channels, such as the tip-localized hyperpolarization-activated Ca^2+^ channels. These Ca^2+^ channels are induced by D-myo-inositol-1,4,5-triphosphate (IP3), which is formed by phospholipase C (PLC) in the PLC-IP3 pathway (Kost et al., 1999; Qu et al., 2017). PLC cleaves phosphatidylinositol 4,5-bisphosphate (PIP2) to IP3 to stimulate influx of extracellular free Ca^2+^ through Ca^2+^ channels. In *Pyrus pyrifolia*, S-RNase interacts with PLC in an allele-specific manner. In case of the presence of self S-RNase, this interaction will result in a severe inhibition of PLC, while the presence of non-self S-RNase in the same concentration will only cause a minimal inhibition. When the activity of pollen tube PLC is blocked by self S-RNase, the concentration of IP3 decreases at the tube apex and Ca^2+^ influx is lowered. This lowered Ca^2+^ influx eventually disrupts the internal Ca^2+^ gradient and hence leads to a reduced growth rate of the incompatible pollen tube (Qu et al., 2017).

A second mechanism causing a reduction of pollen tube growth may be the change in inorganic pyrophosphate (PPi) concentration in the pollen tube (Figure 4). A recent study in apple revealed that S2-RNase physically interacts with soluble inorganic pyrophosphatase (MdPPa), resulting in a non-competitive inhibition of its activity in self-(incompatible) pollen tubes. PPase functions in the removal of excess PPi, hence RNase-based decrease of MdPPA activity leads to elevated levels of inorganic pyrophosphate (PPi). This is associated with an inhibition of tRNA aminoacylation, resulting in accumulation of uncharged tRNA (Li et al., 2018). Uncharged tRNAs regulate global gene expression in response to changes in amino acid pools, as evidenced in bacterial cells, and hence act as effector molecules that stall protein synthesis (Raina and Ibba, 2014). Considering the continuous release of PPi during pollen tube growth, it is now assumed that RNase-based decrease of PPA activity leads to an excess of PPi and uncharged tRNA. This in turn causes cessation of pollen tube elongation (DeGraaf et al., 2006) due to inhibition of the overall cell metabolism (Chen et al., 1990; Li et al., 2018).

A third cellular process occurring during SI-based pollen tube growth inhibition is the depolymerization of filamentous actin in the pollen tube and formation of punctate actin foci (Figure 4) (Liu et al., 2007). S-RNases of *Pyrus bretschneideri* directly interact with filamentous actin (F-actin) in a non-allele-specific manner and depolymerize the actin cytoskeleton in pollen tubes independent of its RNase activity (Chen et al., 2018b). In contrast, apple S-RNases do not directly interact with F-actin. Instead, they inhibit an actin-binding protein complex that contains myosin, villin, and GRAM (MdMVG), and that directly binds to and severs F-actin (Yang et al., 2018). This subtle difference indicates that the actual cellular mechanism of self-pollen tube rejection might differ somewhat between *Malus* and *Pyrus* species. The pollen tube cytoskeleton is essential for both pollen tube growth and transport of sperm cells (Ushijima et al., 2003). Depolymerization of F-actin may therefore in itself be sufficient enough to inhibit pollen tube growth and even cause induction of PCD (Thomas et al., 2006). In the early stages of the self-incompatibility reaction, F-actin depolymerization may be slowed down by the action of phosphatic acid (PPA) (Chen et al., 2018b). This is supported by a study in *Pyrus bretschneideri* that revealed an increased expression of phospholipase D (*Pbr*PLDδ1) upon challenge by self S-RNases (Chen et al., 2018b). Specific knockdown of *Pbr*PLDδ1 thereby accelerated pollen tube death during the early stages of SI and this acceleration was alleviated by the addition of exogenous PPA during SI. It was concluded that an increase in *Pbr*PLDδ1-derived PPA temporarily delays actin depolymerization in the pollen tube and provides a protective mechanism against PCD signaling until sufficient accumulation of incompatible S-RNase ultimately triggers induction of PCD. Following F-actin depolymerization, intracellular transglutaminases (TGases) are proposed to induce aberrant reorganization of F-actin in incompatible pollen tubes to form the observed actin foci (Poulter et al., 2011). In *Pyrus*, the activity of these intracellular TGases has been found to increase in the pollen tubes during the SI response (Iorio et al., 2012).

The processes described above are all linked to the disruption of tip-localized ROS which typically occurs in incompatible pollen tubes following challenge with self S-RNase (Wang et al., 2010). In compatible pollination events, ROS generated by NADPH oxidases in the mitochondria and plasma membrane accumulate at the pollen tube tip to form a gradient that promotes pollen tube elongation (Potocký et al., 2007). In line with this, compatible pollen tubes show an accumulation of mitochondria together with an accumulation of H_2_O_2_ in the mitochondria and the cell wall of the subapical region of the pollen tube. Upon challenge with self S-RNase, the mitochondrial membrane potential collapses, causing cytosolic leakage of cytochrome c and disruption of ROS production in incompatible pollen tubes (Figure 4). In line with this, self S-RNase-treated pollen tubes in *Pyrus pyrifolia* showed a complete lack of H_2_O_2_ in the mitochondria and cytosol (Wang et al., 2009, 2010). Additionally, the presence of self S-RNase in the pollen tube was found to substantially reduce NADPH levels, causing a decrease in ROS formation at the plasma membrane (Figure 4) (Wang et al., 2010). These events were all observed *in vitro* and occur immediately after addition of self S-RNase to the *Pyrus pyrifolia* pollen tubes. Moreover, when an NADPH oxidase inhibitor (diphenylene iodonium chloride, DPI) and a ROS scavenger (TMPP) are used to mimic ROS disruption, the same events occur as observed in the presence of S-RNase: decreased Ca^2+^ currents, depolymerized actin cytoskeleton, and induction of nuclear DNA degradation.

These results indicate that tip-localized ROS disruption occurs very early in the SI response in *Pyrus*, and putatively acts as a central trigger for pollen tube growth inhibition and PCD (Wang et al., 2010). However, as described above, S-RNase also directly interacts with several other targets, to cause decreased Ca^2+^ currents, increase in PPi levels, and actin depolymerization independent of ROS. It is therefore still unclear which sequence of events makes up the SI response. Moreover, as most of these analyses were performed using *in vitro* systems, described observations and their timing may be artificial or differ somewhat from the actual events occurring during SI response in the pollen tube.

### Compatible Pollen in *Pyrus* GSI: Recognition of Non-self S-RNases and Their Degradation

The first step in the SI response of *Pyrus* is the uptake of S-RNase protein by the pollen tube from the transmitting tissue of the style in a non-allele specific way. Both self and non-self S-RNases enter the pollen tube (Luu et al., 2000; Goldraij et al., 2006; Meng et al., 2014b), indicating that the self-recognition process happens inside the pollen tube and that there is a mechanism that inhibits the activity of non-self S-RNases, but not of self S-RNases (Figure 4) (DeFranceschi et al., 2012). S-RNase uptake by the pollen tube has been proposed to occur *via* two ways: by endocytosis (Luu et al., 2000; Goldraij et al., 2006; Meng et al., 2014b) or by membrane transporters (Meng et al., 2014a; Williams et al., 2015). In apple, evidence for both processes exists (Meng et al., 2014a,b). *In vitro* tests showed that S-RNase uptake by the pollen tube depends on Golgi vesicle trafficking and additionally relies on an intact and dynamic cytoskeleton (Meng et al., 2014b). In parallel, the membrane-bound ATP-binding cassette transporter MdABCF was found to promote transport of S-RNase into the apple pollen tube (Meng et al., 2014a). MdABCF is thereby thought to coordinately interact with the cytoskeleton to support S-RNase import into the pollen tube. After uptake, the S-RNases are recognized as being self or non-self. For GSI in *Pyrus*, a recognition model was proposed based on two important findings. The first finding was the identification of an F-box gene as the pollen S-determinant in the Rosaceae (Lai et al., 2002; Entani et al., 2003; Qiao et al., 2004), suggesting that S-RNases are recognized by an F-box protein in the pollen tube, marking them for degradation by the 26S proteasome (Zhang et al., 2009; DeFranceschi et al., 2012). However, the large number of *S-RNase* allele variants and their high degree of sequence diversity raised the question of how one single F-box protein can specifically mark all non-self S-RNases, but leave the self S-RNase intact (Williams et al., 2015). This was explained by a second important finding, i.e., the concomitant presence of multiple SFFB genes on the S-locus in both *Pyrus* and *Malus* species (Sassa et al., 2007). Each of these SFBB proteins could recognize a single or multiple non-self S-RNases and multiple SFBB proteins can work together to interact with a single non-self S-RNase. The SFBB protein that specifically recognizes the self S-RNase would in that case never be present in the S-haplotype, leaving the self S-RNase untargeted and free to inhibit growth of the self (incompatible) pollen tube. Importantly, a phenomenon observed earlier in both Rosaceae and Solanaceae supports this model, i.e., competitive interaction. Competitive interaction occurs when a single pollen grain is universally self- and cross-compatible, because it carries two different S-locus haplotypes (heteroallelic pollen). This occurs, for example, in diploid pollen produced by polyploid varieties. Because in this situation, all the pollen grains carry two different haplotypes (e.g., S1-S2), there is no incompatibility possible because pollen can degrade both types of self S-RNases. More specifically, the S1-RNase of the style is degraded by the action of the SFBB genes present on the S2-haplotype, while the S2-RNase is targeted by the SFBB genes of the S1-haplotype (DeFranceschi et al., 2012). In contrast, pollen carrying two copies of the same S-haplotype (homoallelic pollen) remains self-incompatible (Crane and Lewis, 1942; Lewis, 1947; Brewbaker, 2010; Qi et al., 2011b). Remarkably, *Prunus* only harbors one *F-box* gene as pollen S-determinant and shows full absence of competitive interaction (Hauck et al., 2005; Nunes et al., 2006). This has led to the assumption that there exist two different regulatory models for GSI in the Rosaceae, namely “self-recognition by a single factor” in the *Prunus* genus and “non-self-recognition by multiple factors” in the *Pyrus* and *Malus* genera (Kao and McCubbin, 1996; Sassa et al., 2007; Kubo et al., 2010; DeFranceschi et al., 2012).

In short, the accepted model for *Prunus* species proposes that the pollen-expressed SFB protein protects self S-RNases from degradation by the general inhibitor (GI) which binds non-specifically to all S-RNases in the pollen tube. In case of an incompatible reaction, the SFB protein recognizes the enzyme complex consisting of the GI and the self S-RNase and polyubiquinates the GI for degradation. This will protect and release the cytotoxic self S-RNase and eventually lead to degradation of self-pollen tube (incompatible). In case of a compatible reaction, the SFB protein does not recognize the non-self S-RNase and GI enzyme complex and the non-self S-RNase remains inhibited by the GI (Ushijima et al., 2003; Tao and Iezzoni, 2010; Matsumoto and Tao, 2016a). Protein interaction analysis and *in vitro* ubiquitination assays revealed that the three SLFLs in the *Prunus* S-locus interact both with SSK1 (SLF-interacting Skp1-like protein 1) and S-RNase and can tag ubiquitin molecules onto the S-RNases (Chen et al., 2018a). This finding suggests that the three SLFLs are likely candidates for the GI (Matsumoto and Tao, 2016b; Chen et al., 2018a). The SLFLs show close relationship with the SFBB genes of *Pyrus* (Aguiar et al., 2015; Akagi et al., 2016) and may hence degrade S-RNase in a very similar manner (Matsumoto and Tao, 2016b). However, more evidence is needed to unambiguously confirm that the SLFLs act as the GI.

In contrast, the “non-self-recognition by multiple factors” model of *Pyrus* assumes multiple pollen S-determinants (SFBBs) that function in a non-self-recognition system for S-RNase degradation (Sassa et al., 2007; Kubo et al., 2010). A functional S-haplotype is proposed to possess multiple SFBBs that each recognize and inhibit a single or a subset of non-self S-RNases, but to lack the SFBB protein(s) that recognize the self S-RNase. Self S-RNases are therefore not inhibited and hence cause the rejection of incompatible pollen tubes (Kubo et al., 2010). In this aspect, the GSI model for *Pyrus* is much more similar to the model for Solanaceae compared to that for *Prunus* (DeFranceschi et al., 2012).

Recently, molecular studies provided more insights into the specific interaction of SFBBs with non-self RNase. In *Pyrus*, recognition of self/non-self S-RNase is proposed to happen through direct interaction with one or several SFBBs in the cytosol of the pollen tube (Li et al., 2016). In *Pyrus* and *Malus*, the RHV and possibly four other protein regions (PS1–PS4) of the S-RNase play an important role in this specific recognition (Vieira et al., 2007; Li et al., 2016).

### Degradation of Non-self S-RNases in *Pyrus* GSI: Mechanistic Insights

**Figure 5:**
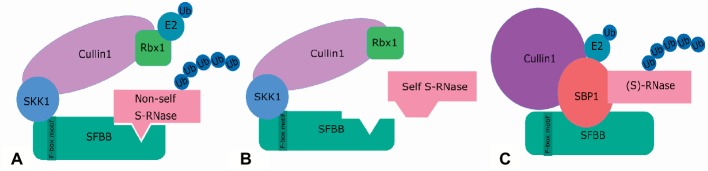
Two SCF^SLF^ complexes are proposed to operate concomitantly in the recognition of S-RNases in the pollen tube of Rosaceae and Solanaceae: the SCF^SLF^ complex **(A,B)** and the SBP1-containing complex **(C)**. **(A)** The SCF^SLF^ complex is considered the main agent in self/non-self S-RNase discrimination in both Rosaceae and Solanaceae. It consists of an F-box protein (SFBB in Rosaceae), which determines the allele-specific interaction with the S-RNase, a pollen-specific Cullin 1, SSK1, and Rbx1. When a non-self S-RNase is recognized by the F-box protein, the S-RNase is ubiquitinated by the E2-conjugating enzymes, marking it for degradation by the 26S-proteosome. **(B)** When the S-RNase is not recognized, no interaction will occur and therefore no ubiquitination, leaving the self S-RNases intact. **(C)** The SBP1-containing complex is proposed to mediate a basal level of S-RNase degradation. This complex acts in a non-S-allele-specific manner and contains S-RNase-binding protein (SBP1) instead of Skp1 and Rbx1, together with a different Cullin1 protein. In this complex, SBP1 is suggested to replace the function of RBX1 and SSK1.

The actual detoxification of non-self S-RNases during a compatibility reaction in *Pyrus* is currently described by two models: the “Protein Degradation Model” and the “Compartmentalization Model” (McClure et al., 2011). The “Protein Degradation Model” proposes ubiquitination and subsequent degradation by the 26S proteasome of all non-self S-RNases after interaction with one or multiple SFBB proteins (Hua et al., 2008; McClure et al., 2011). S-RNase degradation is thereby mediated by an SCF complex, which generally consists of four components: an F-box protein, Skp1, Cullin1, and Rbx1 (Xu et al., 2013). The F-box protein determines substrate specificity, and Skp1 connects the F-box protein to Cul1, which together with Rbx1 transfers a ubiquitin moiety from the ubiquitin-charged E2 enzyme to the substrate (Matsumoto and Tao, 2016a). In *Pyrus bretschneideri*, the complex is referred to as the SLF-containing SCF complex (SCF^SLF^ complex) and contains the SFBB protein, a pollen-specific SSK1 protein (SLF-interacting Skp1-like protein 1), a pollen-specific Cullin1, and Rbx1, as illustrated in Figure 5 (Zhao et al., 2010; Xu et al., 2013; Williams et al., 2015). In Solanaceae, Plantaginaceae, and *Maloidae*, SSK1 interacts with the pollen-specific F-box protein to form an SCF complex (Huang et al., 2006; Zhao et al., 2010; Xu et al., 2013; Li et al., 2014; Minamikawa et al., 2014; Yuan et al., 2014; Matsumoto and Tao, 2016a), supporting a similar mechanism of S-RNase ubiquitination by the SCF complex in *Pyrus*. However, in *Petunia inflata,* another E3 ubiquitin ligase and SCF-like complex, i.e., containing S-RNase-binding protein 1 (SBP1) instead of Skp1 and Rbx1, also seems to be involved in GSI. SBP1 thereby interacts with SLF, another Cul1, as well as with S-RNase, although in a non-allele-specific manner (Figure 5) (Hua and Kao, 2006, 2008; Minamikawa et al., 2014). These interactions, however, are not nearly as strong as with SSK1. The SBP1-containing SCF-like complex therefore probably mediates merely a basal level of S-RNase degradation (Williams et al., 2015). In addition, ubiquitination by the SBP1-containing complex *in vitro* does not occur in an S-allele-specific manner, nor shows specificity to S-RNases (Hua and Kao, 2006). Interestingly, apple also contains a similar pollen-expressed homolog of SBP1, namely MdSBP1 (Yuan et al., 2014). Both MdSBP1 and MdSSK1 interact with MdSFBB *in vitro*, but MdSSK1 interacts more strongly with MdSFBB and its transcript level is over 100 times higher than MdSBP1 (Minamikawa et al., 2014; Williams et al., 2015), suggesting that in apple also, SFBB-mediated degradation of S-RNase predominantly involves MdSSK1. In support of this, MdSBP1 is not specifically expressed in pollen, indicating that it is involved not solely in pollen tube rejection but also in other processes (Yuan et al., 2014).

The “Compartmentalization Model,” on the other hand, is based on findings in tobacco (*Nicotiana alata*) and explains an alternative mechanism for S-RNase detoxification (Goldraij et al., 2006). In this model, both self and non-self S-RNases are taken up by the pollen tube and immediately stored in vacuoles, preventing cytotoxic activity and allowing pollen tube growth through the style. In case of incompatibility, these vacuoles are ruptured and S-RNase is released into the cytoplasm, resulting in inhibition of pollen tube growth. This vacuolar rupture is proposed to happen *via* the action of a non-S-RNase named HT-B after the clone HT from which it was purified (McClure et al., 1999). HT-B is a pistil-specific non-S-factor that was identified in *Nicotiana* and *Solanum*, and that is stabilized by a complex of self S-RNase and SLF (Goldraij et al., 2006). Evidence of sequestration of S-RNases into the pollen tube vacuoles was also found in *M. domestica* (Meng et al., 2014b). It is thereby assumed that S-RNase proteins entering the pollen tube are enveloped by Golgi-derived vesicles which subsequently transport them *via* actin and microtubule filaments to the vacuole for sequestration. However, strong evidence supporting this is as yet lacking.

It is possible that a combination of S-RNase “protein degradation” and “compartmentalization” describes the actual sequence of events in pollen recognition of *Pyrus*, as is proposed in Solanaceae (Williams et al., 2015). In that case, the SCF^SLF^ complex, described above, may mono-ubiquitinate non-self S-RNases in the cytosol to mark them for transport to the vacuole (Shenoy, 2016), while it may also poly-ubiquitinate non-self S-RNases for direct degradation. In case of self S-RNase, the SCF-like complex cannot bind, but the non-S-RNase-specific SCF-like complex carrying SBP1 may mediate S-RNase poly-ubiquitination for baseline degradation or mono-ubiquitination for vacuolar sequestration. However, in the latter case, the majority of the self S-RNases are expected to remain intact and, when concentration of self S-RNase increases, tolerance of the pollen tube will be trespassed leading to pollen tube growth abortion (Williams et al., 2015). In Solanaceae, the unification of the two models might explain two contradicting observations: (1) the requirement of SCF^SLF^ complexes in the cytosol of the pollen tube for a compatible reaction and (2) the sequestration of the S-RNases into the vacuole (Williams et al., 2015). In contrast, in *Pyrus*, the degradation model is more readily accepted as the sole model for pollen tube recognition.

### Breakdown of Self-Incompatibility Allows Self-Fertilization

Transition to self-compatibility is commonly observed in self-incompatible species. This suggests that the benefits of producing outbred offspring of higher genetic quality do not always outweigh the drawbacks of a reduced progeny in cases where pollination or the availability of compatible pollen donors is limited (Vallejo-Marín, 2007; Igic et al., 2008; Baldwin and Schoen, 2017). Induction of self-compatibility (SC) in otherwise self-incompatible (SI) plants may result from either physiological or genetic changes, including mutations (DeNettancourt, 1977). Physiological changes leading to self-compatibility are always temporary and are collectively referred to as pseudo-compatibility (PC). In most species, individuals are either self-compatible (SC) or self-incompatible (SI), however the situation seems to be more complex in *Pyrus* (Sanzol and Herrero, 2007). European pear cultivars have been classified as either completely SI, weakly SC, or completely SC, however, with strong dependency on environmental conditions (e.g. temperature) and year-to-year variations. This has resulted in contradictory reports for the same cultivar (Griggs and Iwakiri, 1954; Callan and Lombard, 1978; Sanzol and Herrero, 2007; Moriya et al., 2009). Both in pear and apple, the strength of the SI response varies depending on different intrinsic and extrinsic parameters, including tree or flower age, flower quality, ambient temperature, and application of plant hormones (DeNettancourt, 1997). Self-incompatibility in pear and apple can also be overcome by specific pollination techniques, such as the use of mentor pollen (i.e., a mixture of compatible and incompatible pollen) or pioneer pollen (i.e., pollination with compatible, but sterilized pollen followed by self-pollination) (Visser, 1981). Finally, cultivar differences in self-(in)compatibility may also be caused by differences in S-RNase expression and abundance in the style (Hiratsuka and Zhang, 2002).

Induction of self-compatibility in SI species can also originate from alterations at the genetic level. Three distinct types of genetic changes are relevant, namely: induction of polyploidy, modification of S-locus incompatibility gene(s), and genetic modification of non-S-locus factors.

Polyploidy in *Pyrus* and *Malus* results in pollen-determined self-compatibility through competitive interaction in its S-heteroallelic diploid pollen, as described above (Crane and Lewis, 1942; Adachi et al., 2009). Breakdown of SI due to alterations in the DNA sequence of incompatibility genes can be classified into two types: modifications in the S-RNase and modifications in the pollen S-gene. Several *Pyrus* genotypes exhibit breakdown of SI due to a mutation in the pistil S-gene. The Japanese pear variety “Osa-Nijisseiki” is a naturally occurring self-compatible mutant. This mutant variety harbors two different S-haplotypes (*Pp*S2-*Pp*S4); however, it lacks the complete *PpS4-RNase* gene due to a deletion of more than 4 kb spanning the entire length of the *PpS4-RNase* gene (Sassa et al., 1997). In Chinese pear (*Pyrus bretschneideri*), the variety “Yan Zhuang,” a self-compatible sport of the “Ya Li” variety, was identified as a pistil-part mutant that is caused by a point mutation in the 182^nd^ nucleotide of the *PbrS21-RNase* sequence. The resulting Gly-to-Val substitution significantly affects the stability of the S-RNase leading to self-compatibility (Li et al., 2009). In European pear (*Pyrus communis*), genotyping of the *S-RNase* alleles of the self-compatible varieties “Abugo” and “Ceremeno” led to the identification of a mutation in *PcS121*, which is referred to as PcS121*. This new allele has a 561-nt retrotransposon insertion within the intron together with two indels of 2 and 30 bp at the 3’UTR region which could explain the absence of PcS121* gene expression in styles of both “Abugo” and “Ceremeno” (Sanzol, 2009a). Mutations in the female S-locus determinant that do not lead to SC but give rise to distinct, although functionally identical, variants of the same *S-RNase* allele are also possible. Genomic analysis of *S-RNase* sequences of 28 European pear cultivars led to the identification of two distinct variants of the *PcS104*-allele. These variants differ at five nucleotide positions, but do not confer functional difference as in both cases self-incompatibility is maintained. Interestingly, two of these SNPs lead to an alteration of the predicted protein sequence, without affecting the corresponding pollen or pistil SI function (Sanzol, 2010). These results suggest that the different *S-RNase* sequences, i.e., referred to as *PcS104–1* and *PcS104–2*, represent transitional states in the process of generating new *S-RNase* alleles (Sanzol, 2010).

While many self-compatible pollen-part mutants are known in *Prunus* (Ushijima et al., 2004; Beppu et al., 2005; Sonneveld et al., 2005; Vilanova et al., 2006), breakdown of SI due to genetic defects in the pollen S-determinant is not expected to occur in *Pyrus* due to the non-self-recognition system of SI. Mutations leading to non-functional *SFBB* genes inherently imply a full absence of S-RNase degradation (both in self and non-self interactions), leading to the targeted degradation of otherwise compatible pollen tubes and cross-incompatibility with all other S-genotypes. For example, the previously mentioned pistil-compatible mutant “Osa-Nijisseiki” has a large deletion that not only spans the *PpS4-RNase* but also includes the *SFBB* gene immediately upstream of *PpS4-RNase*. Deletion of this *SFBB* gene not only renders the pollen self-incompatible (inhibited growth on PpS4-styles), but also cross-incompatible with PpS1-genotypes (inhibited growth on PpS1-styles) (Okada et al., 2008). For a long time, the only known true pollen-part self-compatible mutants (PPMs) in *Pyrus* were polyploid, because of the occurrence of competitive interaction. However, several years ago, the first pollen-part mutant (PPM) in a diploid *Pyrus* variety was identified, namely the variety 415–1 of Japanese pear (*Pyrus pyrifolia*). This line was produced by fertilizing “Kosui” with pollen from gamma-irradiated “Kosui” with *S-RNase* genotype *Pp*S4*Pp*S5 (Sawamura et al., 2013). Although being diploid, this variety exhibits a segmental duplication that encompasses the complete S5-haplotype block. As a result, the *Pyrus pyrifolia* variety 415–1 produces S-heteroallelic pollen containing both S4- and S5-haplotype blocks which can penetrate S4-S5 styles because of competitive interaction (Mase et al., 2014).

Breakdown of SI in *Pyrus* has also been reported to occur in the absence of mutations in the *S-RNase* or *SFBB* genes. This suggests that induction of SC can also be caused by the alteration of non-S-locus factors. In Chinese pear (*Pyrus bretschneideri*), the self-compatible cultivar “Zaoguan” (*Pbr*S4-*Pbr*S34) accepts self-pollen in its styles. However, the S-locus genes are free of genetic defects and the pollen is rejected in a normal manner on styles of other incompatible pear cultivars. Transcriptional analysis revealed a full absence of *PbrS34-RNase* expression in “Zaoguan” pistils, indicating that the self-compatibility is caused by a yet unknown alteration in the transcriptional regulation of *Pbr*S34-RNase (Qi et al., 2011a). However, loss of *S-RNase* expression may also be caused by epigenetic alterations in the promoter or open reading frame (ORF) of the *S-RNase* sequence. Two other examples of self-compatibility caused by unknown non-S-locus factors were found in the Chinese pear (*Pyrus bretschneideri*) variety “Jin Zhui,” i.e., another self-compatible sport of “Ya-li,” and in the Japanese pear (*Pyrus pyrifolia*) variety “XinXue” (Li et al., 2009; Wu et al., 2013; Shi et al., 2018). The unknown non-S-locus factor underpinning self-compatibility in “Jin Zhui” was recently suggested to be the *PLC* gene. As previously described, the S-RNase interacts in an allele-specific way with PLC to inhibit its activity. This action results in a decreased activity of Ca^2+^ channels at the pollen tube tip and thus disrupts the internal Ca^2+^ gradient in the pollen tube. The *PLC* gene of “Jin Zhui” shows a 26-amino acid insertion and no longer interacts with self S-RNase, suggesting that self-compatibility in the “Jin Zhui” variety is attributed to functional loss of PLC (Qu et al., 2017).

## Practical Aspects of GSI in *Pyrus*: Pollination and Fruit Set

### Implications of Self-Compatibility for Pear Fruit Development

It is considered most favorable for the production of high-quality fruit that all ovules of the flower are fertilized. Developing seeds release plant hormones, such as auxins, that cause ovary expansion, so that the fruit mainly grows and expands at regions where fertilized seeds are located (Devoghalaere et al., 2012; Orcheski and Brown, 2012). In case only a subfraction of the ovules is fertilized, resulting fruits can be small (Westwood, 1993; Goldway et al., 2008). Pollination must therefore lead to fertilization. In theory, incompatible pollen tubes are inhibited while compatible pollen tubes are allowed to grow through the style. In practice, however, actual inhibition of pollen tubes is not only controlled by the genetic determination, but is also influenced by several external factors, such as the environment and number of pollination events. Studies in pear and apple have described the positive effect of multiple pollination events on selfed seed set in self-incompatible lines (Visser and Marcucci, 1984). The application of two consecutive pollination events generally leads to an increased seed set, and this increase seems to vary depending on the compatibility of each pollination. Self-pollination before cross-pollination (S/C) produces more seeds in incompatible pear varieties than cross-pollination before self-pollination (C/S), and both produce more seeds than a single cross-pollination event (Visser and Verhaegh, 1980; Visser, 1981; Visser et al., 1983; Zimmerman, 1988). This phenomenon is known as “pioneer pollen effect,” in which a previous pollination event facilitates pollen tube growth during a second pollination event (Visser, 1981). A similar phenomenon is known as “mentor pollen” where an equal mixture of self- and cross-pollen produces a fair amount of selfed seed (Visser, 1981; Montalti and Filiti, 1984). In practice, open pollination in pear generally results in a very low number of pollen deposited on the style due to low abundance and activity of insect pollinators (Konarska et al., 2005; Jacquemart et al., 2006). Repeated pollination events are rare under natural conditions because insect pollinators are unlikely to revisit a flower (Giurfa, 1996; Witjes and Eltz, 2007; Wilms and Eltz, 2008). Moreover, in pear cultivation, the pollen mixture that reaches the stigma of *Pyrus* SI species mainly consists of self-pollen, particularly considering the fact that insect pollinators in pear orchards typically visit multiple flowers of the same tree (Visser and Marcucci, 1984; Pannell and Labouche, 2013). It is therefore expected that pollen interactions such as mentor or pioneer pollen do not frequently occur under natural conditions in *Pyrus*. Interestingly, when self-pollen does fertilize the ovule, ovule abortion is higher than in case of cross-pollination, suggesting the additional presence of one or more post-zygotic reproductive barriers that block formation of seed upon selfing (Martin and Lee, 1993). Seed abortion is influenced by the pollen source and in case of self-pollen, this abortion may be due to homozygous recessive lethal alleles resulting from selfing (Martin and Lee, 1993). It is therefore proposed that self-seeds with non-lethal, but inferior allele combinations are more prone to abortion (Martin and Lee, 1993; Pannell and Labouche, 2013). However, it is not excluded that selfed seeds have lower sink strength compared to those resulting from an outcrossing event, and hence show higher level of seed and/or fruit abortion due to competition for energy acquisition.

### Self-Incompatibility in Pear Production and Breeding

The SI mechanism in pear dictates that fruit set in most commercial cultivars strongly depends on successful cross-pollination and fertilization, hence posing major implications for both commercial pear production and breeding. In order to guarantee fruit set, commercial pear orchards need to contain at least two cross-compatible cultivars or combine the commercial cultivar with a pollen donor variety, such as a wild pear species. Moreover, both cultivars involved should exhibit overlapping flowering periods to enable effective cross-fertilization, i.e., to enable seed set which in its turn stimulates fruit development (Goldway et al., 2012). The identification and knowledge of the exact S-genotype of different pear varieties is hence crucial for many practical applications, including orchard design and the success of hybridization crosses in pear breeding programs (Sanzol and Robbins, 2008).

Overall, there are three scenarios for (in)compatibility of diploid cultivars of *Pyrus*: (1) when two different parent cultivars carry identical S-haplotypes, they are fully incompatible; (2) when they share only one of their S-haplotypes, they are semi-compatible; and (3) when they differ in both S-haplotypes, they are fully compatible. Although semi-compatibility does not affect fruit set rate in hand-pollination experiments, it can cause significant reductions in fruit yield when environmental conditions are suboptimal for pollination (Schneider et al., 2005; Zisovich et al., 2005; Goldway et al., 2008; Sapir et al., 2008). From a practical point of view, however, a complete lack of cross-pollination is not entirely problematic in some pear varieties. Firstly, several pear varieties, such as “Conference,” exhibit a natural potency of parthenocarpy and hence do not require pollination for induction of fruit set (Nishitani et al., 2012). Such varieties do not require fertilization and hence produce seedless fruits. Alternatively, parthenocarpy can also be induced by application of hormones, such as gibberellins. However, despite the promising role of pollination-independent fruit set, parthenocarpic pear varieties often produce fruit that is smaller compared to that resulting from cross-pollination, making them less suitable for commercial fruit production (Nishitani et al., 2012). Secondly, self-fertilization due to pseudo-compatibility has repeatedly been documented in several *Pyrus* varieties. However, successful self-fertilization in these cases is expected to vary considerably between seasons and cultivars (Williams et al., 1994). Moreover, similar as in parthenocarpic varieties, pear fruit resulting from self-fertilization is generally smaller and is more likely to abscise early compared to that resulting from cross-pollination events (Atwell et al., 1999).

In pear breeding applications, the intercrossing of two fully incompatible varieties is impossible as all pollen is rejected. Two incompatible pear varieties can only be intercrossed and hybridized *via* the use of specific techniques, such as mentor pollen, gamma irradiation of pollen, cut style techniques, or polyploidization (Atwell et al., 1999). In a similar way, crosses between two semi-incompatible varieties may also cause problems. In such crossing events, all pollen with a specific S-genotype is rejected, leading to a limited number of possible S-genotype combinations in the offspring. This “artificial selection” has a significant impact on the diversity of S-alleles in commonly grown cultivars, and leads to a reduced genetic and biological diversity in cultivated pears. Specific cultivars, like for example “Williams Bon Chrétien” (or “Bartlett”), are frequently used as a parent for the development of new cultivars. As a consequence, their corresponding S-alleles are overrepresented in newly developed commercial cultivars (Sanzol and Robbins, 2008; Orcheski and Brown, 2012). Interestingly, “Williams Bon Chrétien” carries the S-alleles *Pc*S101 and *Pc*S102, while most of its selected descendants carry the *Pc*S101-allele and not the *Pc*S102-allele. This shows that the *PCS101-*allele is favored during selection, suggesting that the S-locus is linked to one or more genes that underpin important traits for pear cultivation or fruit quality (Sanzol and Robbins, 2008). This intrinsically means that interesting traits may be lost in semi-incompatible crosses because pollen carrying the common S-allele will be rejected. In contrast, self-incompatibility can also have advantages. For example, SI can be handy when doing crosses, because female parents do not need to be emasculated before being pollinated by the desired male parent (Denna, 1971).

## Conclusion and Future Perspective

Over the years, many studies have provided insights into the self-incompatibility mechanism of *Pyrus.* For example, the identification and molecular characterization of the pollen-part S-determinant has been a major focus during recent years. Furthermore, the identification of multiple *SFBB* genes, which are linked to the S-locus, has strengthend the hypothesis that pollen tubes are recognized according to “the non-self recognition” mechanism with multiple factors, similar to some Solanaceae species. However, it is still unclear how many *SFBB* genes are present and which of the identified SFBBs are actually involved in the non-self-recognition system. In addition, much is still unknown about the structure of the S-locus, more specifically, the positioning of the *SFBB* genes around the *S-RNase*, and its variability between varieties or different pear species. The characterization of natural, self-compatible mutants contributes further to our knowledge of the GSI mechanism in *Pyrus*, especially those mutants that confer self-compatibility through still uncharacterized, but S-locus-linked factors.

The unraveling of the molecular mechanism(s) underlying pollen tube rejection in *Pyrus* GSI has gained increasing attention in fundamental research. The discovery that the S-RNase can interact with multiple targets besides the pollen S-determinant has revealed a multifactoral role for the S-RNase in the self-incompatibility reaction with several other functions besides RNA degradation. Based on growing evidence, it is likely that a much more complex mechanism underpins the rejection of self-pollen tubes in *Pyrus*. Finally, the characterization of natural, self-compatible mutants contributes to our knowledge of the genetic control and molecular regulation of GSI in *Pyrus*. Particularly, mutants conferring SC through uncharacterized, but S-locus-linked factors may provide new insights into the complex regulation of GSI in *Pyrus*. Such knowledge can be useful, for example, in the development of self-compatible varieties through convential breeding or by using gene editing techniques, like CRISPR-Cas9.

As self-incompatibility affects fertilization, seed set, and fruit quality in pear orchards and has important implications for pear production and breeding, it is essential that research keeps exploring its underlying mechanisms. Ultimately, new insights into pear self-incompatibility can result in new and targeted applications that may facilitate pear production and breeding.

## Author Contributions

NDS, WK, BvDP and HC contributed to the main conceptual ideas and manuscript outline. HC wrote the manuscript with inputs, corrections and critical feedback from the other authors.

### Conflict of Interest Statement

The authors declare that the research was conducted in the absence of any commercial or financial relationships that could be construed as a potential conflict of interest.
